# Prevalence of diabetic retinopathy and its associated factors among adults in East African countries: A systematic review and meta-analysis

**DOI:** 10.1371/journal.pone.0316160

**Published:** 2025-01-31

**Authors:** Habtamu Wagnew Abuhay, Tigabu Kidie Tesfie, Meron Asmamaw Alemayehu, Muluken Chanie Agimas, Getaneh Awoke Yismaw, Gebrie Getu Alemu, Nebiyu Mekonnen Derseh, Bantie Getnet Yirsaw

**Affiliations:** Department of Epidemiology and Biostatistics, Institute of Public Health, College of Medicine and Health Sciences, University of Gondar, Gondar, Ethiopia; Eye Foundation Hospital / Eye Foundation Retina Institute, NIGERIA

## Abstract

**Introduction:**

Diabetes mellitus (DM) is one of the most significant public health problems. Globally, one in ten adults has diabetes, and it results in macro- or microvascular complications, such as diabetic retinopathy (DR). It is one of the most prevalent eye complications associated with DM, and it is the main cause of vision loss. Even though East African countries face a growing burden of diabetes and DR, no study depicts the regional prevalence and its associated factors. Therefore, this study aimed to estimate the pooled prevalence of DR and its associated factors among adults in East African countries.

**Methods:**

We extensively searched PubMed, Embase, Scopus, Google Scholar, and Google for relevant studies. A forest plot was used to estimate the pooled prevalence of diabetic retinopathy using DerSimonian and Laird’s random-effects model. We checked publication bias using funnel plots and Egger’s regression test. Potential heterogeneity was tested using the I-squared statistic. Subgroup analysis, sensitivity analysis, and meta-regression analysis were also performed. Furthermore, the pooled odds ratios for the associated factors were estimated. The research protocol was registered in PROSPER.

**Results:**

Among the 29 included studies, the estimated pooled prevalence of DR in East African countries was 28% (95% CI 23.0, 33.0). Besides, age ≥60 (OR = 2.88, 95% CI: 1.55, 5.32), body mass index ≥ 25 (AOR = 2.85; 95% CI: 1.69, 4.81; I2 = 85.4%, p < 0.001), and hemoglobin A1c levels ≥7 (OR = 2.48, 95% CI: 1.46, 4.23) were significantly associated with the prevalence of DR.

**Conclusions:**

The prevalence of DR in East Africa was high, with more than one in four individuals with diabetes developing DR. Besides, advanced age, higher body mass index, and elevated hemoglobin A1c levels were significant factors associated with increased DR prevalence. Therefore, comprehensive diabetes management focusing on optimal glycemic control and healthy weight maintenance is essential to mitigate the problems. Also, the Ministries of Health and policymakers should prioritize and implement targeted strategies to address the identified modifiable risk factors, aiming to reduce the prevalence of DR in the region.

**Trial registration:**

**Systematic review registration:** PROSPERO (2024: ID = CRD42024511437). https://www.crd.york.ac.uk/prospero/display_record.php?ID=CRD42024511437.

## Introduction

Diabetes mellitus (DM) is a chronic metabolic disorder characterized by elevated blood sugar levels [1]. Diabetes results from either insufficient insulin production by the pancreas or the inability of the body cells to utilize the insulin that is produced [2]. It is currently one of the most significant public health problems in the world [3]. In 2021, approximately 537 million adults around the globe (one in ten adults) had diabetes [4], with the majority residing in low- and middle-income countries [5].

DM is characterized by elevated blood glucose levels that lead to widespread damage to the vascular system [6]. It might result in macro- and microvascular complications, such as neuropathy, nephropathy, and retinopathy [6,7]. Diabetic retinopathy (DR) is one of the most prevalent eye-related complications associated with diabetes mellitus [8]. It appears when high blood sugar levels damage the blood vessels in the retina, and it is the main cause of vision loss [8,9].

Globally, it is estimated that 27.0% of patients with diabetes have DR [10], In In Africa, the prevalence of DR ranges from 7.0% to 62.4%[11], and in East Africa, the reported prevalence of DR ranges from 13% to 82.6% [12]. Different studies showed that age, sex, duration of diabetes, types of diabetes, glycemic levels, body mass index, and comorbidity status (hypertension), as well as diabetes-related complications such as diabetic neuropathy and diabetic nephropathy, have been associated with the occurrence of DR [13–16].

Although East African countries face a growing burden of diabetes and DR, there are no representative data that show the extent of the magnitude of the problem and its related factors in these regions. Therefore, this study aimed to estimate the pooled prevalence of diabetic retinopathy and its associated factors among adults in East African countries. Addressing this issue can provide valuable insights for developing targeted public health measures, implementing effective strategies, and strengthening healthcare systems.

## Methods and materials

### Reporting

This systematic review and meta-analysis was performed and, reported in accordance with the Preferred Reporting Items for Systematic Review and Meta-Analysis Statement (PRISMA 2020) guidelines [17]. In addition, the protocol for this study was registered in the International Prospective Register of Systematic Reviews (PROSPER) with a protocol number (ID = CRD42024511437).

### Inclusion and exclusion criteria

To establish inclusion and exclusion criteria, we applied the CoCoPop (Condition, Context, and Population) framework for the prevalence studies. The criteria were as follows:

### Condition

Studies addressed the prevalence and associated factors of DR.

### Context

Studies conducted in East African countries (Burundi, Comoros, Djibouti, Eritrea, Ethiopia, Kenya, Madagascar, Malawi, Mauritius, Mozambique, Rwanda, Seychelles, Somalia, South Sudan, Tanzania, Uganda, Zambia, and Zimbabwe) at the hospital or health facility level.

### Population

Adults aged 18 years and above who had DM were considered. In addition, both published and unpublished observational studies, conducted between January 1, 2015, and January 31, 2024, and written in English, were included. However, studies of patients with DM other than type-1 and type-2, studies without full-text availability, and qualitative studies were excluded.

### Search strategy and information sources

Databases, such as PubMed, Embase, Scopus, and Google Scholar, were comprehensively searched for potential studies. In addition, we searched Google for gray literature and reference lists of other previously identified studies. The basic search terms and phrases were prevalence, diabetic retinopathy, associated factors, and East African countries.

Following this, the MeSH headings, synonyms, and search terms (Boolean operators) were identified and searched as ((("Prevalence" OR "Prevalence"[Mesh] OR "magnitude" OR "incidence" OR "Incidence" [Mesh])) AND (("diabetic retinopathy" OR "Diabetic Retinopathy"[Mesh] "diabetic eye complication" OR "diabetic macular edema" OR "diabetic angiopathy" OR "diabetic angiopath?"))) AND ((Burundi OR Comoros OR Djibouti OR Eritrea OR Ethiopia OR Kenya OR Madagascar OR Malawi OR Mauritius OR Mozambique OR Rwanda OR Seychelles OR Somalia OR South Sudan OR Tanzania OR Uganda OR Zambia OR Zimbabwe)). We searched for publications published between January 1, 2015, and January 31, 2024. See **(S1 Table).**

### Study selection process and quality assessment

Duplicate studies were eliminated by exporting the retrieved studies to the reference manager program, EndNote version 9. Then studies were reviewed based on their title and abstract by four independent authors (TKT, GAY, NMD, and GGA). Disagreements were resolved by applying the agreed-upon article selection criteria and consulting with the fifth author (HWA). Then, full-text reviews were conducted by three authors (HWA, MCA, and BGY), and articles that did not meet the eligibility criteria were excluded along with reasons. The eligibility of all retrieved studies was assessed by the authors (HWA, MAA, MCA, and BGY) using the Joanna Briggs Institute (JBI) quality appraisal checklist [18]. Finally, studies that scored 50% on or above on the quality assessment checklist criteria were considered high quality and included in this study **(S2 and S3 Tables).**

### Measurement of the outcome variable

This systematic review and meta-analysis had two outcome variables. The first outcome was the prevalence of DR, defined as a complication of diabetes affecting the blood vessels in the retina [19], and is characterized by progressive microvascular change such as aneurysm, intra-retinal edema, and intraocular pathologic neovascularization [20].

The second outcome was the factors associated with DR. We identified these factors and used odds ratios (ORs) to measure the strength of the association between diabetic retinopathy and its predictors.

### Data extraction

A standardized data extraction form was prepared in a Microsoft Excel 2016 spreadsheet and utilized. Three authors (HWA, NBD, and MAA) independently extracted relevant data using the following variables from the included studies: first author name, study country, year of publication, study design, year of data collection, sample size, sex, age of the study participant, prevalence of DR, type of diabetes, duration of diabetes, hemoglobin A1C level, body mass index, and adjusted odds ratio (AOR) of associated factors. Disagreements and inconsistencies among the authors regarding the extracted data were addressed through discussion and by repeating the extraction procedures **(S1 File).**

### Statistical analysis

To assess publication bias in this systematic review and meta-analysis, a funnel plot and Egger’s regression test were employed. A p-value of 0.05 in Egger’s regression indicated the presence of publication bias [21]. The heterogeneity among the studies was assessed using the I-squared statistic, with thresholds of 25%, 50%, and 75% indicating low, moderate, and high heterogeneity, respectively [22]. For the pooled analysis of DR, random-effects DerSimonian-Laird models were utilized. Subgroup analysis was conducted based on the publishing country, publication years, and study design. Finally, sensitivity analyses and meta-regression analyses were performed to determine the influence of a single study on the overall estimates and to identify the sources of heterogeneity.

### Ethical approval and consent

Ethical approval and consent to participate did not apply to this study, because this is a systematic review and meta-analysis based on primary studies published on diabetic retinopathy in East African countries.

## Results

### Study selection and identification

For this study, a total of 2,577 articles were identified through various electronic databases.

**Fig 1:**
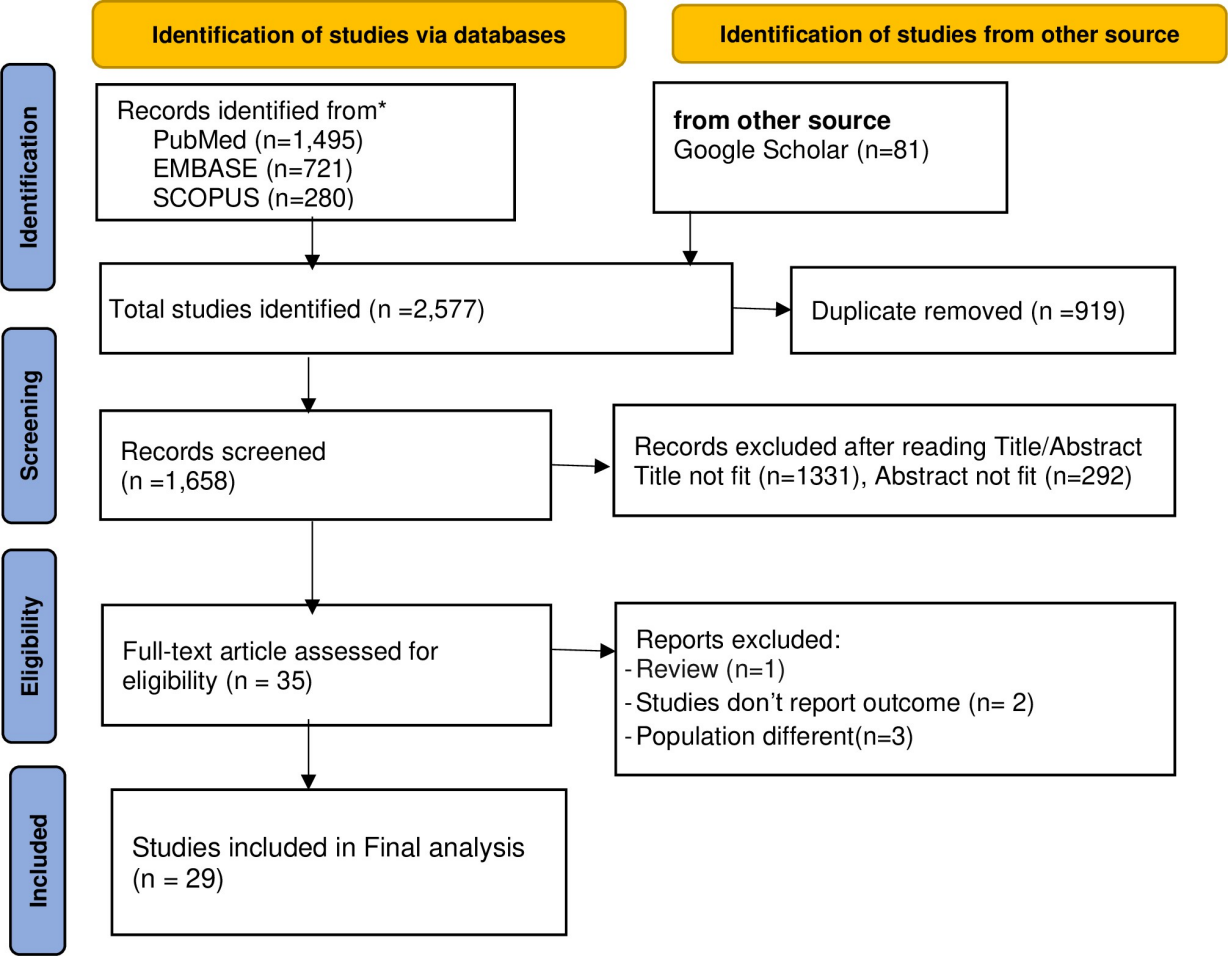
PRISMA 2020 flow diagram for systematic reviews and meta-analysis of diabetic retinopathy and its associated factors among adults in East African countries. This included searches of databases, registers, and other sources.

After removing 919 duplicates, 1,658 articles remained. The titles and abstracts of these articles were screened, resulting in the exclusion of 1,623 articles. Of the 35 articles suitable for full-text review, 6 were excluded based on the predetermined criteria. Finally, 29 primary studies were deemed eligible and included in this systematic review and meta-analysis **(Fig 1).**

### Characteristics of the included studies

A total of 14,687 participants from 29 studies were included in this systematic review and meta-analysis, of whom 4,447 developed DR. All the studies were published between the years 2015 and 2024. The included studies exhibited varying sample sizes, the largest study involved 3467 patients from Tanzania [23], while the smallest study included 87 patients from Uganda [24]. The distribution of studies by country was as follows: five in Kenya [25–29], fourteen in Ethiopia [13–15,30–40], two in Rwanda [41,42], two in Uganda [24,43], One in Malawi [44], Mozambique [45], South Sudan [46], Tanzania [23], Zambia [47], and Zimbabwe [16], were included. See **(Table 1).**

**Table 1:** Characteristics of the included studies and prevalence of diabetic retinopathy in East African Countries, 2024.

S.no	Author	Year	Country	Study design	Sample size	DR	Risk of bias
		2022	Ethiopia	Cross-sectional	331	131	Low
2	Azeze et al.[37]	2018	Ethiopia	Retrospective Cohort	377	70	Low
3	Burgess et al.[44]	2015	Malawi	Cross-sectional	357	178	Moderate
4	Chisha et al.[30]	2017	Ethiopia	Cross-sectional	400	52	Low
5	Cleland et al.[23]	2016	Tanzania	Cross-sectional	3467	967	Low
6	Debele et al.[32]	2021	Ethiopia	Retrospective Cohort	402	81	Low
7	Ejigu and Tsegaw.[14]	2021	Ethiopia	Cross-sectional	255	95	Low
8	Gelcho and Gari.[33]	2022	Ethiopia	Retrospective Cohort	373	154	Low
9	Iradukunda et al.[42]	2021	Rwanda	Cross-sectional	246	64	Low
10	Ireri et al.[25]	2024	Kenya	Cross-sectional	314	34	Low
11	Lewis et al.[47]	2018	Zambia	Cross-sectional	2153	1113	Low
12	Lewis et al.[16]	2022	Zimbabwe	Retrospective Cohort	135	42	Low
13	Magan et al.[24]	2019	Uganda	Cross-sectional	87	17	Moderate
14	MoH Kenya.[29]	2018	Kenya	Cross-sectional	256	64	Low
15	Musawa et al.[26]	2022	Kenya	Cross-sectional	329	102	Low
16	Niyodusenga A et al.[41]	2021	Rwanda	Case-Control	592	67	Low
17	Nyakaba et al.[28]	2023	Kenya	Cross-sectional	149	47	Low
18	Olwendo et al.[27]	2020	Kenya	Retrospective Cohort	489	59	Low
19	Rigato et al.[45]	2022	Mozambique	Retrospective Cohort	536	156	Low
20	Sahiledengle et al.[15]	2022	Ethiopia	Cross-sectional	256	45	Low
21	Seba et al.[43]	2015	Uganda	Cross-sectional	168	28	Low
22	Seid et al.[36]	2021	Ethiopia	Case-Control	282	142	Low
23	Shibru et al.[38]	2019	Ethiopia	Cross-sectional	191	98	Low
24	Sube et al.[46]	2020	South-Sudan	Cross-sectional	108	14	Moderate
25	Takele et al.[35]	2022	Ethiopia	Retrospective Cohort	494	142	Low
26	Tassew et al.[39]	2023	Ethiopia	Retrospective Cohort	403	78	Low
27	Tilahun et al.[31]	2020	Ethiopia	Cross-sectional	302	57	Low
28	Tsegaw et al.[40]	2021	Ethiopia	Cross-sectional	739	200	Low
29	Zegeye et al.[34]	2023	Ethiopia	Cross-sectional	496	180	Low

### Pooled prevalence of diabetic retinopathy in East African countries

**Fig 2:**
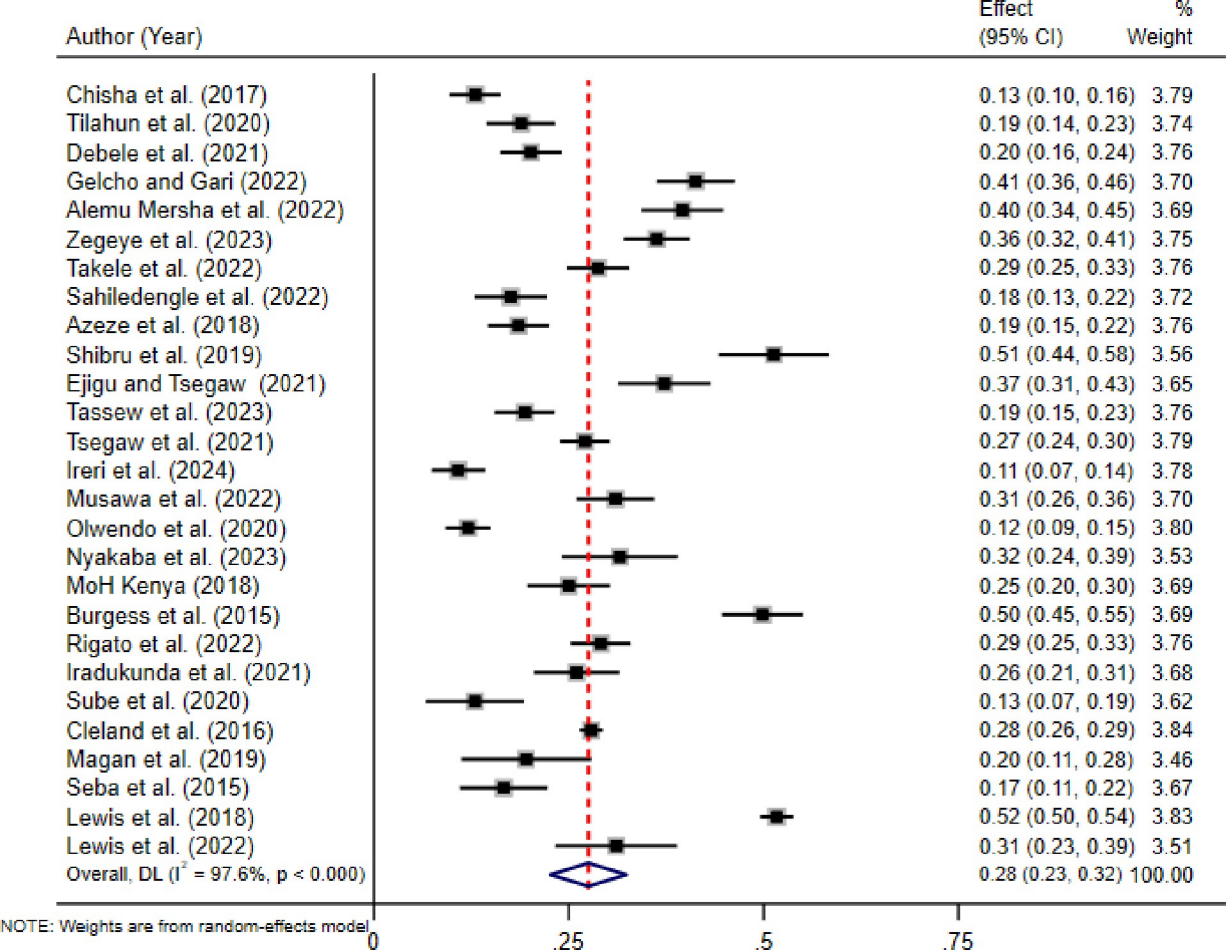
Forest plot of the pooled prevalence of diabetic retinopathy in East African countries, 2024.

In the random-effects model, the overall pooled prevalence of DR in East African countries was 28% (95% CI 23.0, 33.0). Statistically significant heterogeneity was observed among studies (I^2^ = 97.60, p-value < 0.001). The lowest prevalence of DR was observed in a study conducted in Kenya [25], 11% (95% CI 07, 14), while the highest prevalence was observed in a study conducted in Zambia [47], 52% with (95% CI: 50, 54) **(Fig 2).**

### Publication bias

**Fig 3:**
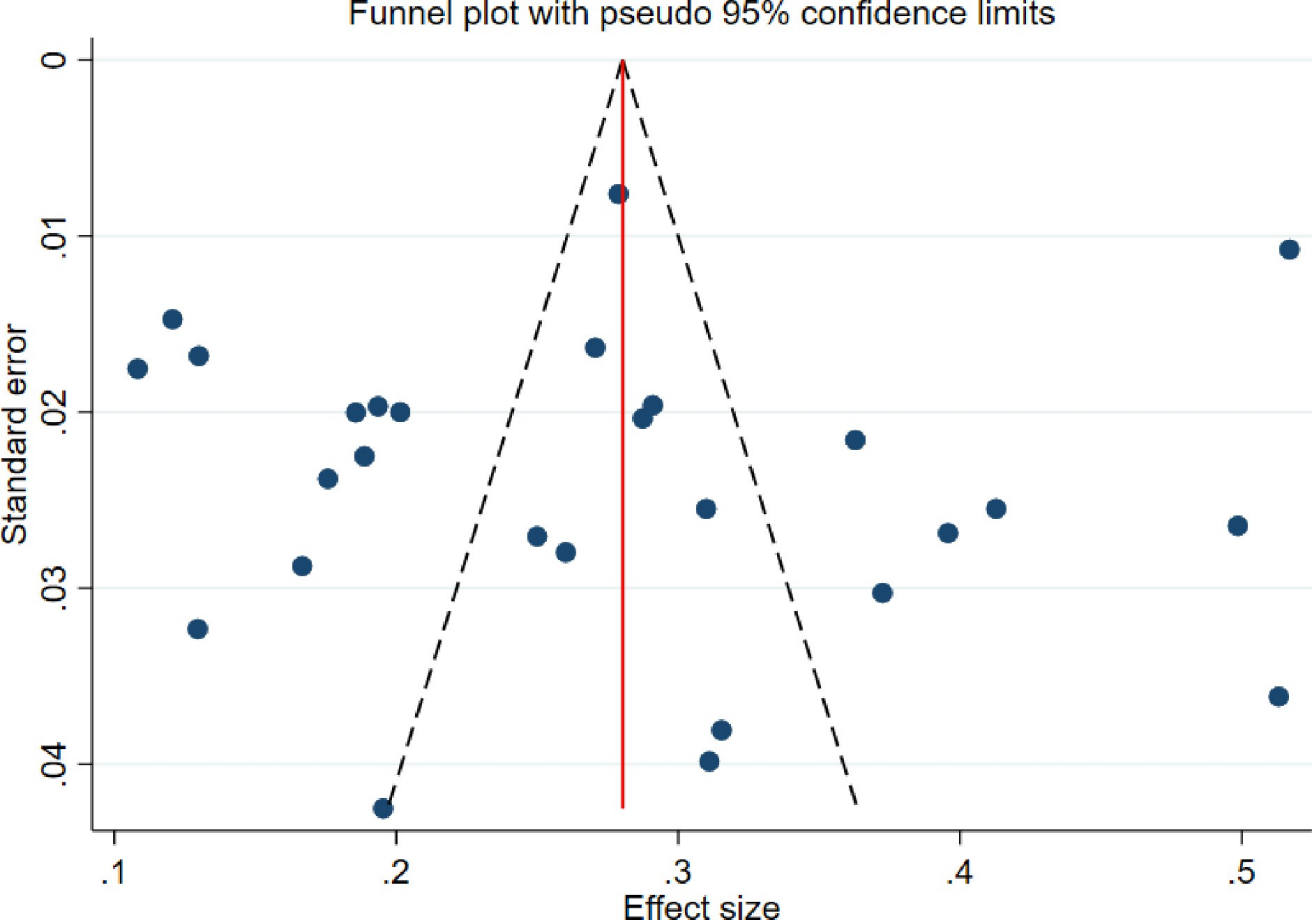
Funnel plot illustrating publication bias for the pooled prevalence of diabetic retinopathy in East African countries.

To assess the presence or absence of publication bias, a funnel plot was visually inspected. The result indicated asymmetry, which may suggest the presence of publication bias **(Fig 3).** However, Egger’s regression test was subsequently performed to objectively confirm publication bias, and the results indicated no significant publication bias (p-value = 0.985) **(S1 Fig).**

### Handling heterogeneity

From the pooled estimate of the random effect model, significant heterogeneity was observed. As a result, subgroup analyses, sensitivity analyses, and meta-regression analyses were performed to address this heterogeneity.

### Subgroup analyses

Subgroup analyses were performed based on the study region (publishing country). The pooled prevalence of DR was estimated to be 28.0% in Ethiopia (95% CI: 23.0, 34.0; I^2^ = 95.45%, p-value < 0.001), which was higher than the pooled prevalence in Kenya, 22.0% (95% CI: 13.0, 30.0; I^2^ = 94.81%, p-value < 0.001) **(S2 Fig).**

In addition, subgroup analyses by publication year were conducted. For studies published between 2015 and 2019, the pooled prevalence of DR was 30.0% (95% CI: 20.0, 41.0; I^2^ = 98.78%, p-value < 0.001), which was higher than the pooled DR prevalence in studies conducted between 2020 and 2024, which was 26.0% (95% CI: 22.0, 31.0; I^2^ = 94.65%, p-value < 0.001) **(S3 Fig).**

Furthermore, subgroup analysis was conducted based on the study design of the primary studies. The pooled prevalence of DR from cross-sectional studies was 29.0% (95% CI: 22.0, 35.0; I^2^ = 97.93%, p-value < 0.001), which was higher than the pooled prevalence from retrospective cohort studies, reported at 25.0% (95% CI: 18.0, 31.0; I^2^ = 94.19%, p-value < 0.001) **(S4 Fig).**

Although subgroup analyses were carried out based on publishing country, publication years, and study designs, the sources of heterogeneity were not addressed. As a result, sensitivity analysis and meta-regression were performed to further investigate these sources.

### Sensitivity analysis

To examine the effect of a single study on the overall prevalence estimates, sensitivity analysis was performed using a random-effect model. The results indicated that no individual study excessively influenced the overall pooled estimate of diabetic retinopathy when studies were alternately omitted from the model **(S5 Fig).**

### Meta-regression

We applied a univariate meta-regression to investigate the source of heterogeneity in the main studies that were included. The analysis revealed no significant associations when sample size, response rates, mean age of study participants, and publication year were used as covariates **(Table 2).**

**Table 2:** Univariate meta-regression analysis results for the pooled prevalence of diabetic retinopathy in East African countries.

Covariates	Coefficients (95% CI)	Standard error	P-value
Mean age	0.003 (-0.084, 0.092)	0.004	0.316
Publication year	0.03 (-0.017, 0.236)	0.010	0.750
Sample size	-0.014 (-0.096, 0.067)	0.041	0.730
Response rates	0.010 (-0.015, 0.021)	0.055	0.053

### Factors associated with diabetic retinopathy among diabetes patients

To identify factors associated with the prevalence of DR in East African countries, a separate random effect pooled estimate analysis was conducted on the extracted factors, including age, sex, residence, types of diabetes, blood pressure, triglyceride, Body mass index (BMI), and blood glucose level. The analysis revealed that age greater than or equal to 60 years, BMI ≥25, and hemoglobin A1c level ≥7 were statistically significantly associated with the prevalence of DR in East African countries **(Table 3).**

**Table 3:** Summary of the pooled effects of factors associated with diabetic retinopathy in East African countries, 2024.

Variable	Category	OR (95% CI)	Heterogeneity (I^2^, P-value)	Egger’s P-value	Total studies	Sample size
Age	< 60 year	1	1			
	≥60 year	2.88(1.55, 5.32)*	61.0%(0.053)	0.266	4	1,105
Sex	Female	1	1			
	Male	1.13(0.96, 1.33)	57.8% (0.002)	0.329	17	9,837
Residence	Urban	1	1			
	Rural	0.81(0.61, 1.08)	61.5% (0.016)	0.073	7	5,694
Types of diabetes mellitus	Type I DM	1	1			
	Type II DM	1.89(0.76, 3.06)	87.9% (0.001)	0.131	9	4,234
Systolic blood pressure	<140mmhg	1	1			
	≥140mmhg	1.10(0.62, 1.77)	94.5% (0.001)	0.032	7	2,398
Triglyceride level	Normal	1	1			
	High	1.97(0.82, 3.25)	81.0% (0.001)	0.047	5	1,984
Body mass index (BMI)	<24.9	1	1			
	≥25	2.85(1.69, 4.81)*	85.4% (0.001)	0.741	8	2,819
Hemoglobin A1c levels	<7	1	1			
	≥7	2.48(1.46, 4.23)*	91.1% (0.001)	0.461	9	3,190

* Statistically significant at 5% level, OR: Odds Ratio, CI: Confidence Interval.

**Fig 4:**
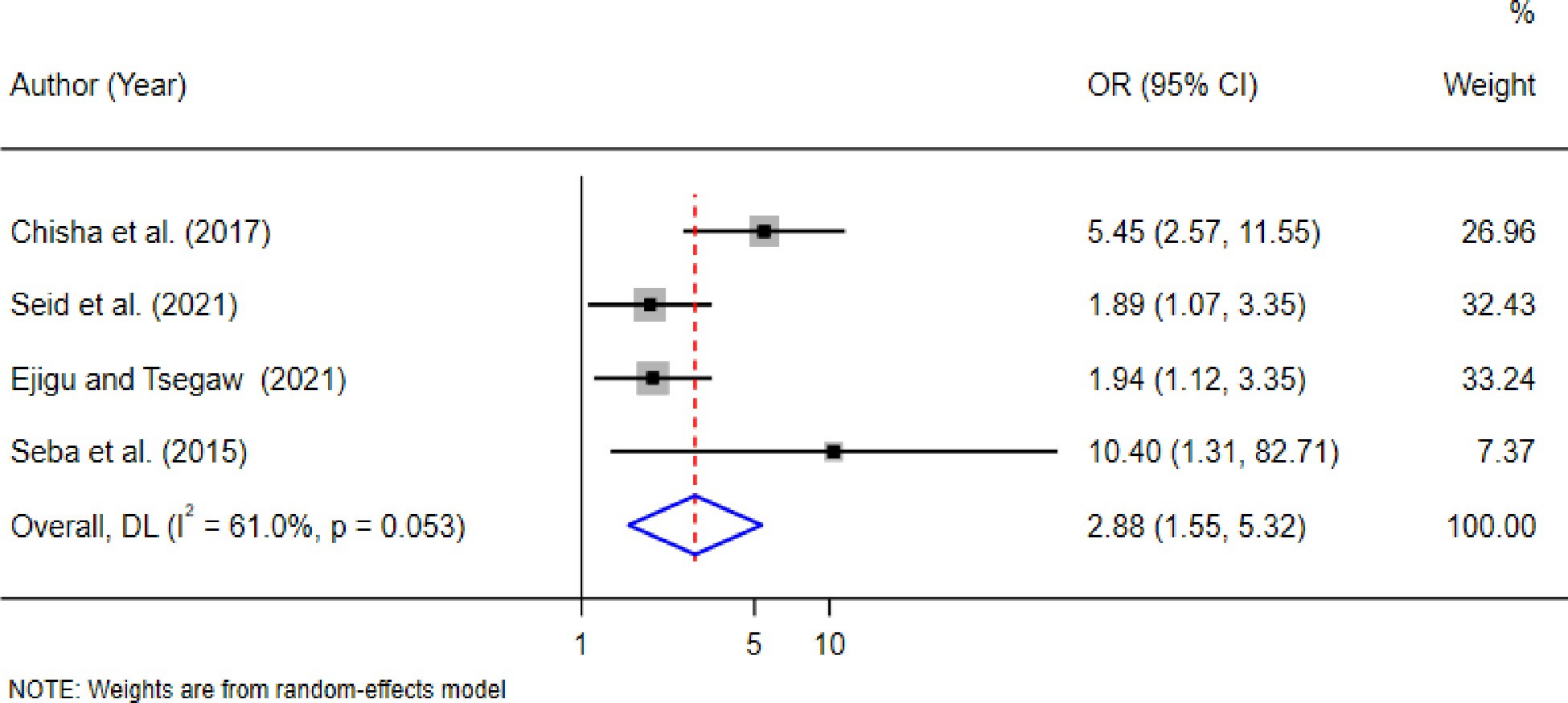
Factors age ≥60 with the prevalence of diabetic retinopathy in East African countries.

Four studies [14,30,36,43], were included to assess the association between age and DR. The random-effects model estimate indicated that the pooled odds of developing DR among individuals aged 60 years and older were 2.88 times higher than those younger than 60 years (OR = 2.88, 95% CI: 1.55, 5.32); the forest plot shows that there was no heterogeneity between studies (I^2^ = 61.0%, P-value < 0.053). See **(Fig 4).**

**Fig 5:**
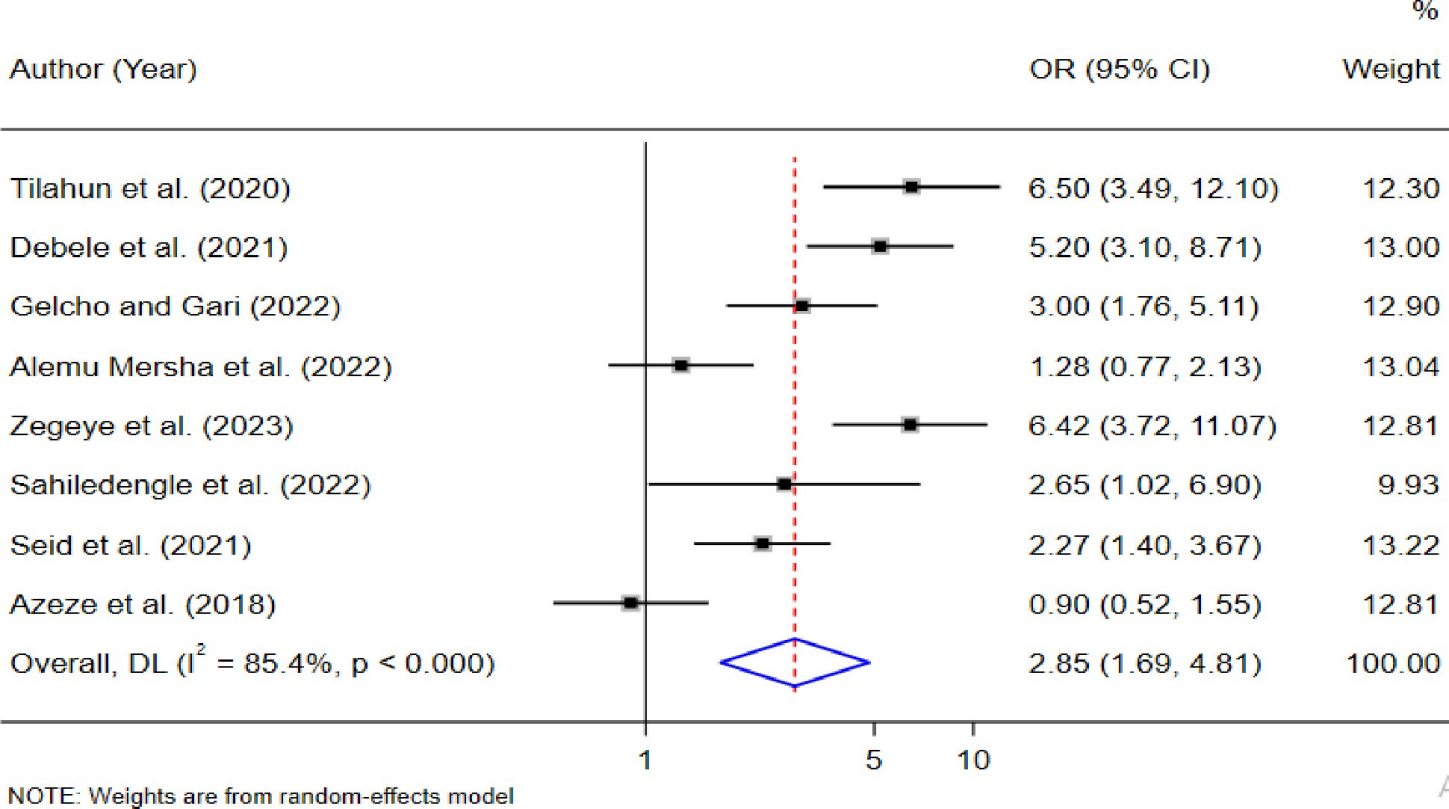
Factors BMI ≥25 with the prevalence of diabetic retinopathy in East African countries.

From the random-effects model of eight studies [13,15,31–34,36,37], a BMI of ≥ 25 was significantly associated with DR. The pooled odds ratio indicated that individuals with a BMI of ≥25 had a 2.85 times higher risk of developing DR compared to their counterparts (AOR = 2.85; 95% CI: 1.69, 4.81; I^2^ = 85.4%, p < 0.001). See **(Fig 5).**

**Fig 6:**
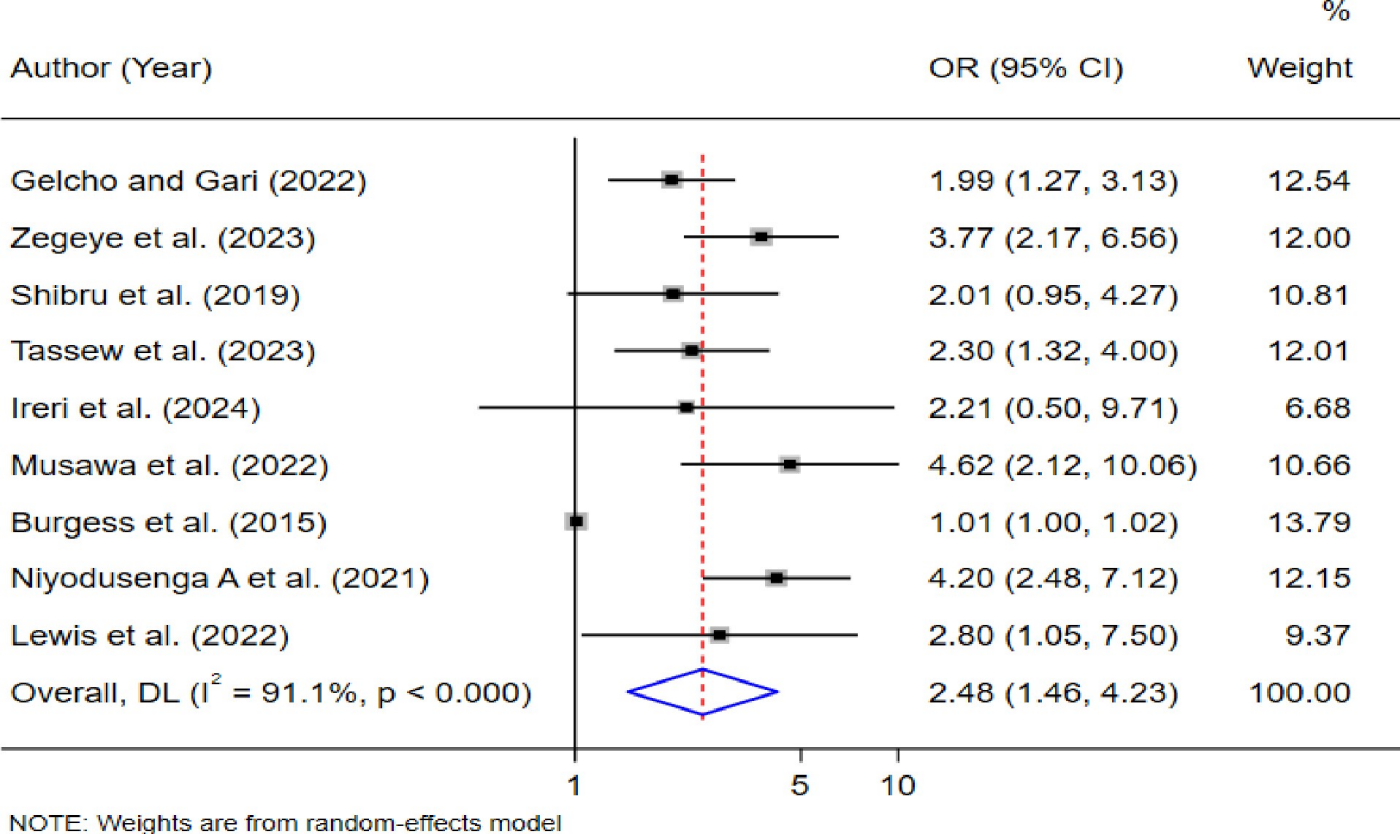
Factors hemoglobin A1c levels ≥7 with the prevalence of diabetic retinopathy in East African countries.

The pooled estimated odds ratio from nine studies [25,26,33,34,38,39,41,44], showed that hemoglobin A1c levels of ≥7 were associated with a 2.48 times higher likelihood of developing DR compared to hemoglobin A1c levels of less than 7 (OR = 2.48; 95% CI: 1.46, 4.23). Statistically significant heterogeneity was observed among the studies (I^2^ = 91.1%, p < 0.001). See **(Fig 6).**

## Discussions

Diabetic retinopathy is a major global public health concern and it is the leading cause of irreversible blindness among adults who have diabetes millions of individuals worldwide are affected by DR, and this number is anticipated to increase as the prevalence of diabetes continues to rise. Notably, the prevalence of DR is rising in developing countries in contrast to developed nations. As a result, this study aimed to provide up-to-date estimates of the pooled prevalence and associated factors of DR in East African countries, which will be of great benefit to policymakers, health planners, and the population as well.

This systematic review and meta-analysis revealed that the pooled estimate of the overall prevalence of DR in East African countries was 28% (95% CI: 23.0, 33.0). This finding is consistent with studies conducted in Asia [48] and the Eastern Mediterranean regions [49], where the estimated prevalence of DR was 28% and 31%, respectively. However, the pooled prevalence in this study is higher than the findings from studies in Spain and China, which reported prevalence’s of 15.28% and 18.45%, respectively [50,51]. These differences may be attributed to variations in the socioeconomic status of DM patients, the quality of health service delivery in the respective countries, the availability of infrastructure and advanced medical services, and the presence of qualified healthcare providers.

In this study, the subgroup analysis revealed a pooled prevalence of DR was 30.0% in studies published between 2015 and 2019, compared to 26.0% in studies conducted between 2020 and 2024. This decline in the prevalence of DR is likely attributed to improved diabetes management, greater access to screenings, and advancements in healthcare service delivery [52,53]. Additionally, increased access to media campaigns raising awareness of diabetes and its complications may have contributed to the reduction in DR prevalence [54].

Additionally, this study identified factors that contribute to the prevalence of DR among diabetes mellitus patients in East African countries. Key factors, such as age greater than or equal to 60, BMI ≥25, and hemoglobin A1c level ≥7, were associated with the prevalence of DR among DM patients. Understanding these factors is essential for developing targeted interventions to improve DR prevention and management.

In this study, age 60 and above was identified as a significant risk factor for DR. This result aligns with findings from both global and regional studies on DR, which have consistently shown a marked increase in DR prevalence after the age of 60 [55]. Similarly, research conducted in Jordan demonstrated that the prevalence of DR significantly rises with advancing age [56]. A possible explanation for this association is that aging is inherently linked to a decline in cellular regeneration and functional capacity [57]. In the retina, this decrease in regenerative ability can impair cell turnover, heightening vulnerability to DR [58]. Additionally, older patients often have a longer history of diabetes, leading to cumulative damage from prolonged hyperglycemia, oxidative stress, and inflammation [59,60]. These combined factors significantly heighten the risk of developing and progressing DR in older adults.

The pooled results from these studies indicate that a BMI of 25 or higher is significantly associated with the occurrence of DR. This finding aligns with a study conducted in Croatia [61], which identified substantial correlations between elevated BMI and the prevalence of DR. Similarly, a study in China found that BMI was notably higher among patients with DR compared to those without the condition [62]. This is because a high BMI is a warning sign of hypertension [63], which contributes to vascular damage throughout the body, including in the retina [64]. Elevated blood pressure intensifies the risk of micro-aneurysms, hemorrhages, and other retinal complications, increasing susceptibility to DR. Therefore, managing BMI will be a key strategy for reducing the risk and progression of DR in diabetic populations.

In this systematic review and meta-analysis, the pooled odds of developing DR among individuals with hemoglobin A1c levels ≥7 were 2.48 times higher than those with hemoglobin A1c levels < 7. This finding aligns with previous studies conducted in South Africa [65], Denmark [66], and China [51], which also demonstrated that higher HbA1c levels are associated with an increased risk of developing DR. Additionally, evidence suggests that reducing HbA1c levels decreases the likelihood of DR incidence among DM patients [67]. Understanding the correlation between HbA1c and DR is crucial for guiding prevention efforts, enhancing early detection, and ultimately reducing the risk of vision loss and other complications.

This systematic review and meta-analysis has some strengths and limitations. This study synthesized data from multiple East African countries, yielding a robust estimate of the pooled prevalence of DR among DM patients. The low risk of bias in the included studies enhances the generalizability of the findings and contributes new evidence to the existing body of knowledge. Additionally, this study identified key factors associated with the prevalence of DR, which can inform targeted clinical and public health interventions. However, the exclusion of unpublished studies and the reliance on studies published only in English may limit the breadth of data, potentially affecting the comprehensiveness of the findings.

## Conclusions and recommendations

The prevalence of diabetic retinopathy in East Africa is notably high, with more than one in four individuals with diabetes developing DR. Besides, advanced age, higher body mass index, and elevated hemoglobin A1c levels were significant factors associated with increased DR prevalence. Therefore, comprehensive diabetes management focusing on optimal glycemic control and healthy weight maintenance is essential to mitigate DR prevalence. Ministries of Health and policymakers should prioritize targeted strategies that address these modifiable risk factors, thereby reducing both the prevalence and impact of DR in the region.

## Supporting information

S1 Table Search Strategy in Included Databases, for diabetic retinopathy in East African countries. (DOCX)

S2 Table Risk of bias assessment for cross-sectional studies. (DOCX)

S3 Table Risk of bias assessment for retrospective cohort study. (DOCX)

S1 Fig Egger’s regression tests to publication bias for the pooled prevalence of diabetic retinopathy in East African countries. (TIF)

S2 Fig Sub-group analysis by country for the pooled prevalence of diabetic retinopathy in East African countries. (TIF)

S3 Fig Sub-group analysis by years of publication for the pooled prevalence of diabetic retinopathy in East African countries. (TIF)

S4 Fig Sub-group analysis by study design for the pooled prevalence of diabetic retinopathy in East African countries. (TIF)

S5 Fig Sensitivity analysis of included studies in the pooled estimates of diabetic retinopathy in East African countries. (TIF)

S1 File Supplementary data file. (XLSX)
