# Supplementary material for: Prevalence of diabetic retinopathy and its associated factors among adults in East African countries: A systematic review and meta-analysis
Source: PLoS One. 2025 Jan 31;20(1):e0316160. doi: 10.1371/journal.pone.0316160 (PMC11785277; doi:10.1371/journal.pone.0316160)
Supplement: S1 Table — (DOCX) [file pone.0316160.s001.docx]

**Supplementary 1 Table: Search Strategy in included databases for diabetic retinopathy studies in East African countries.**

| **Data bases** | **Search strategy** |
| --- | --- |
| PubMed | ((("Prevalence" OR "Prevalence"[Mesh] OR "magnitude" OR "incidence" OR "Incidence" [Mesh])) AND (("diabetic retinopathy" OR "Diabetic Retinopathy"[Mesh] "diabetic eye complication" OR "diabetic macular edema" OR "diabetic angiopathy" OR "diabetic angiopath?"))) AND ((Burundi OR Comoros OR Djibouti OR Eritrea OR Ethiopia OR Kenya OR Madagascar OR Malawi OR Mauritius OR Mozambique OR Rwanda OR Seychelles OR Somalia OR South Sudan OR Tanzania OR Uganda OR Zambia OR Zimbabwe)) n=1,495  Filters applied: from 2015/1/1 - 2024/1/31 |
| Embase | (('diabetic retinopathy' OR 'diabetic macular edema' OR 'diabetic angiopathy') AND burundi:ab,ti OR comoros:ab,ti OR djibouti:ab,ti OR eritrea:ab,ti OR ethiopia:ab,ti OR kenya:ab,ti OR madagascar:ab,ti OR malawi:ab,ti OR mauritius:ab,ti OR mozambique:ab,ti OR rwanda:ab,ti OR seychelles:ab,ti OR somalia:ab,ti OR 'south sudan':ab,ti OR tanzania:ab,ti OR uganda:ab,ti OR zambia:ab,ti OR zimbabwe:ab,ti) AND [article]/lim AND [english]/lim AND [2015-2024]/py AND 'diabetes mellitus':ab,ti AND 'adult' n= 721 |
| Scopus | "diabetic retinopathy" OR "diabetic macular edema" OR "diabetic angiopathy" AND PUBYEAR > 2014 AND PUBYEAR < 2024 AND ( LIMIT-TO ( AFFILCOUNTRY , "Ethiopia" ) OR LIMIT-TO ( AFFILCOUNTRY , "Kenya" ) OR LIMIT-TO ( AFFILCOUNTRY , "Tanzania" ) OR LIMIT-TO ( AFFILCOUNTRY , "Malawi" ) OR LIMIT-TO ( AFFILCOUNTRY , "Uganda" ) OR LIMIT-TO ( AFFILCOUNTRY , "Mauritius" ) OR LIMIT-TO ( AFFILCOUNTRY , "Zambia" ) OR LIMIT-TO ( AFFILCOUNTRY , "Rwanda" ) OR LIMIT-TO ( AFFILCOUNTRY , "Sudan" ) OR LIMIT-TO ( AFFILCOUNTRY , "Zimbabwe" ) OR LIMIT-TO ( AFFILCOUNTRY , "Mozambique" ) OR LIMIT-TO ( AFFILCOUNTRY , "Madagascar" ) OR LIMIT-TO ( AFFILCOUNTRY , "Djibouti" ) OR LIMIT-TO ( AFFILCOUNTRY , "Somalia" ) OR LIMIT-TO ( AFFILCOUNTRY , "Eritrea" ) OR LIMIT-TO ( AFFILCOUNTRY , "Burundi" ) ) AND ( LIMIT-TO ( DOCTYPE , "ar" ) ) AND ( LIMIT-TO ( LANGUAGE , "English" ) ) AND ( LIMIT-TO ( EXACTKEYWORD , "Article" ) ) n**=** 280 |
| Google scholar | With all of word: prevalence of diabetic retinopathy among adults in east Africa countries  with the exact phrases: diabetic retinopathy prevalence OR incidence  At least one of those word: Burundi Comoros Djibouti Eritrea Ethiopia Kenya Madagascar Malawi Mauritius Mozambique Rwanda Seychelles Somalia South Sudan Tanzania Uganda Zambia Zimbabwe.  Searched n**=**81  Limits: 2015 - 2024 |

Legend: tw: text word, DM: diabetes mellitus, mh: MesH term
