# Supplementary material for: Prevalence of diabetic retinopathy and its associated factors among adults in East African countries: A systematic review and meta-analysis
Source: PLoS One. 2025 Jan 31;20(1):e0316160. doi: 10.1371/journal.pone.0316160 (PMC11785277; doi:10.1371/journal.pone.0316160)
Supplement: S2 Table — (DOCX) [file pone.0316160.s002.docx]

**Supplementary 2 Table: Risk of bias assessment for cross-sectional studies.**

| **ID** | **Author** | **Year** | **C1** | **C2** | **C3** | **C4** | **C5** | **C6** | **C7** | **C8** | **#Total score** | **Quality score** |
| --- | --- | --- | --- | --- | --- | --- | --- | --- | --- | --- | --- | --- |
| 1 | Chisha et al. | 2017 | Y | Y | Y | U | N/A | N/A | Y | Y | 5.5 | Low risk |
| 2 | Tilahun et al. | 2020 | Y | N | Y | Y | N/A | N/A | Y | Y | 6 | Low risk |
| 3 | Alemu Mersha et al. | 2022 | Y | Y | U | Y | N/A | N/A | Y | Y | 5.5 | Low risk |
| 4 | Zegeye et al. | 2023 | Y | Y | Y | N | N/A | N/A | Y | Y | 5 | Low risk |
| 5 | Sahiledengle et al. | 2022 | Y | Y | Y | Y | N/A | N/A | Y | Y | 6 | Low risk |
| 6 | Seid et al. | 2021 | Y | Y | Y | Y | N/A | N/A | Y | Y | 6 | Low risk |
| 7 | Shibru et al. | 2019 | Y | Y | Y | Y | N/A | N/A | Y | Y | 6 | Low risk |
| 8 | Ejigu and Tsegaw | 2021 | Y | N | Y | Y | N/A | N/A | Y | Y | 5 | Low risk |
| 9 | Tsegaw et al. | 2021 | Y | Y | U | Y | N/A | N/A | Y | Y | 5.5 | Low risk |
| 10 | Ireri et al. | 2024 | Y | Y | Y | Y | N/A | N/A | Y | Y | 6 | Low risk |
| 11 | Musawa et al. | 2022 | Y | Y | Y | Y | N/A | N/A | Y | Y | 6 | Low risk |
| 12 | Nyakaba et al. | 2023 | U | Y | Y | N | N/A | N/A | Y | Y | 4.5 | Moderate risk |
| 13 | MoH Kenya | 2018 | Y | Y | Y | Y | N/A | N/A | Y | Y | 6 | Low risk |
| 14 | Burgess et al. | 2015 | Y | Y | Y | Y | N/A | N/A | Y | Y | 6 | Low risk |
| 15 | Niyodusenga A et al. | 2021 | Y | Y | Y | Y | N/A | N/A | Y | Y | 6 | Low risk |
| 16 | Iradukunda et al. | 2021 | Y | Y | Y | Y | N/A | N/A | Y | Y | 6 | Low risk |
| 17 | Sube et al. | 2020 | Y | Y | Y | U | N/A | N/A | Y | Y | 5.5 | Low risk |
| 18 | Cleland et al. | 2016 | Y | Y | Y | Y | N/A | N/A | Y | Y | 6 | Low risk |
| 19 | Magan et al. | 2019 | Y | N | Y | Y | N/A | N/A | Y | Y | 5 | Low risk |
| 20 | Seba et al. | 2015 | Y | Y | Y | Y | N/A | N/A | Y | Y | 6 | Low risk |
| 21 | Lewis et al. | 2018 | Y | Y | Y | Y | N/A | N/A | Y | Y | 6 | Low risk |

Y = Yes; N = No; U – Unclear; N/A - Not/Applicable

C1: Were the criteria for inclusion in the sample clearly defined? C2: Were the study subjects and the setting described in detail? C3: Was the exposure measured in a valid and reliable way? C4: Were objective, standard criteria used for measurement of the condition? C5: Were confounding factors identified? C6: Were strategies to deal with confounding factors stated?

C7: Were the outcomes measured in a valid and reliable way? C8: Was appropriate statistical analysis used?

Summarizing Scores

“Yes” was given a value of 1, the score “No” was given a value of 0, the score “Unclear” was given a value of 0.5, and “Not applicable was denoted as N/A

Interpreting the Scores

Low risk of bias (High quality) was nominated if the study scored 6 and above, moderate risk of bias (Good quality) if the study scored between 5 and 4, and High risk of bias (lower quality) for studies scored below 3.
