# Supplementary material for: Prevalence of diabetic retinopathy and its associated factors among adults in East African countries: A systematic review and meta-analysis
Source: PLoS One. 2025 Jan 31;20(1):e0316160. doi: 10.1371/journal.pone.0316160 (PMC11785277; doi:10.1371/journal.pone.0316160)
Supplement: S3 Table — (DOCX) [file pone.0316160.s003.docx]

**Supplementary 3 Table: Risk of bias assessment for retrospective cohort study**

| **ID** | **Author** | **Year** | **C1** | **C2** | **C3** | **C4** | **C5** | **C6** | **C7** | **C8** | **C9** | **C10** | **C11** | **#Total score** | **Quality score** |
| --- | --- | --- | --- | --- | --- | --- | --- | --- | --- | --- | --- | --- | --- | --- | --- |
| 1 | Debele et al. | 2021 | Y | Y | Y | N/A | N/A | Y | Y | Y | Y | Y | Y | 9 | Low risk |
| 2 | Gelcho and Gari | 2022 | Y | U | Y | N/A | N/A | Y | Y | Y | Y | U | Y | 8 | Low risk |
| 3 | Takele et al. | 2022 | Y | U | Y | N/A | N/A | Y | Y | Y | Y | Y | Y | 9 | Low risk |
| 4 | Azeze et al. | 2018 | Y | Y | Y | N/A | N/A | Y | Y | Y | Y | Y | Y | 9 | Low risk |
| 5 | Tassew et al. | 2023 | Y | U | U | N/A | N/A | Y | Y | Y | Y | N | Y | 8 | Low risk |
| 6 | Olwendo et al. | 2020 | Y | Y | Y | N/A | N/A | Y | Y | Y | Y | Y | Y | 9 | Low risk |
| 7 | Rigato et al. | 2022 | Y | Y | U | N/A | N/A | Y | Y | Y | Y | Y | Y | 8 | Low risk |
| 8 | Lewis et al. | 2022 | Y | U | Y | N/A | N/A | Y | Y | Y | Y | Y | Y | 8 | Low risk |

Y = Yes; N = No; U – Unclear; N/A - Not/Applicable

C1: Were the two groups similar and recruited from the same population? C2: Were the exposures measured similarly to assign people to both exposed and unexposed groups? C3: Was the exposure measured in a valid and reliable way? C4: Were confounding factors identified? C5: Were strategies to deal with confounding factors stated? C6: Were the groups/participants free of the outcome at the start of the study (or at the moment of exposure)? C7: Were the outcomes measured in a valid and reliable way? C8: Was the follow up time reported and sufficient to be long enough for outcomes to occur? C9: Was follow up complete, and if not, were the reasons to loss to follow up described and explored? C10: Were strategies to address incomplete follow up utilized? C11: Was appropriate statistical analysis used?

Summarizing Scores

“Yes” was given a value of 1, the score “No” was given a value of 0, the score “Unclear” was given a value of 0.5, and “Not applicable was denoted as N/A

Interpreting the Scores

Low risk of bias (High quality) was nominated if the study scored 8 and above, moderate risk of bias (Good quality) if the study scored between 7 and 5.5, and High risk of bias (lower quality) for studies scored below 5.
