# Supplementary figures and images for: Prevalence of diabetic retinopathy and its associated factors among adults in East African countries: A systematic review and meta-analysis
Source: PLoS One. 2025 Jan 31;20(1):e0316160. doi: 10.1371/journal.pone.0316160 (PMC11785277; doi:10.1371/journal.pone.0316160)

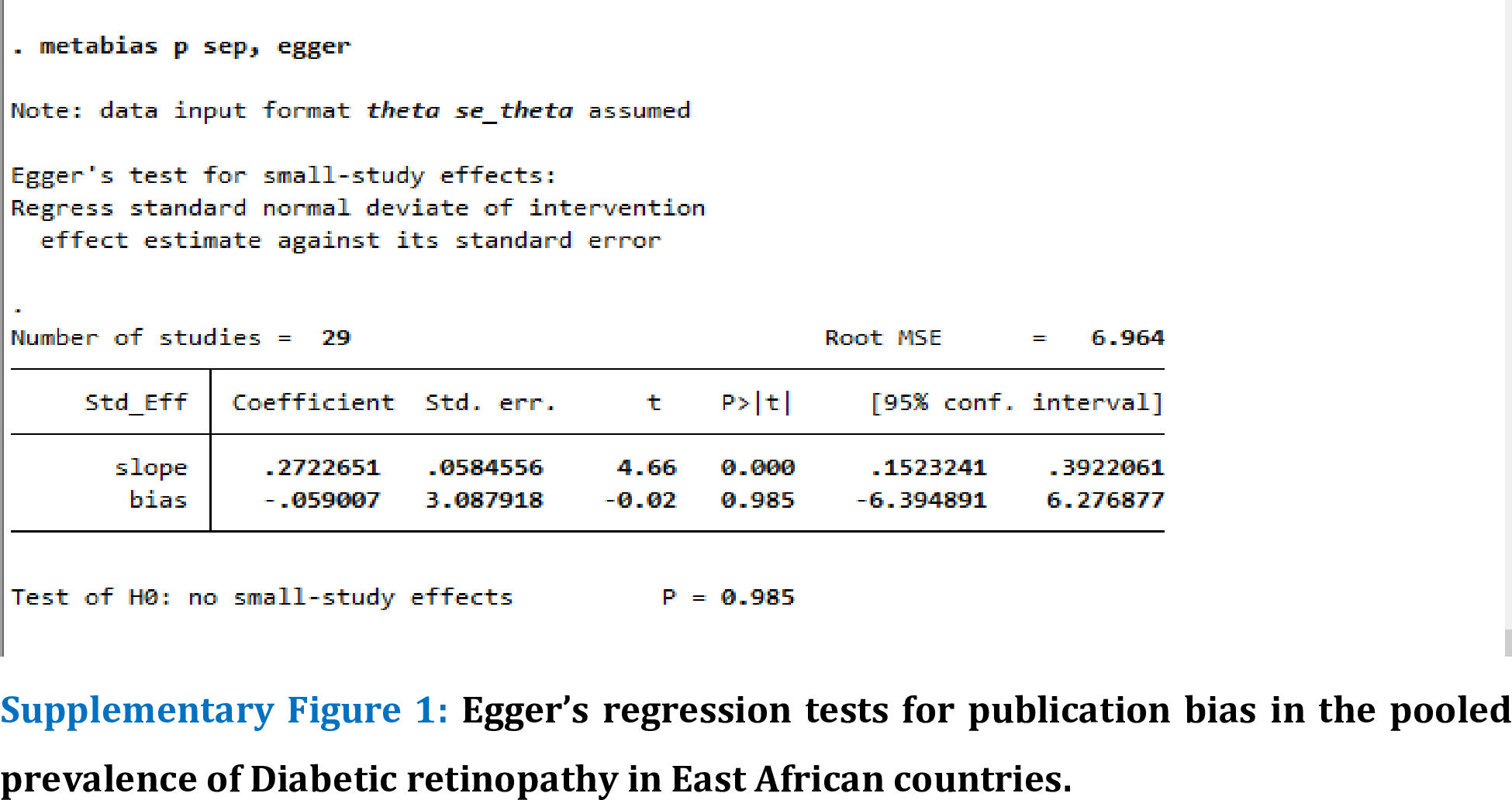

Supplement: S1 Fig — (TIF) [file pone.0316160.s004.tif]

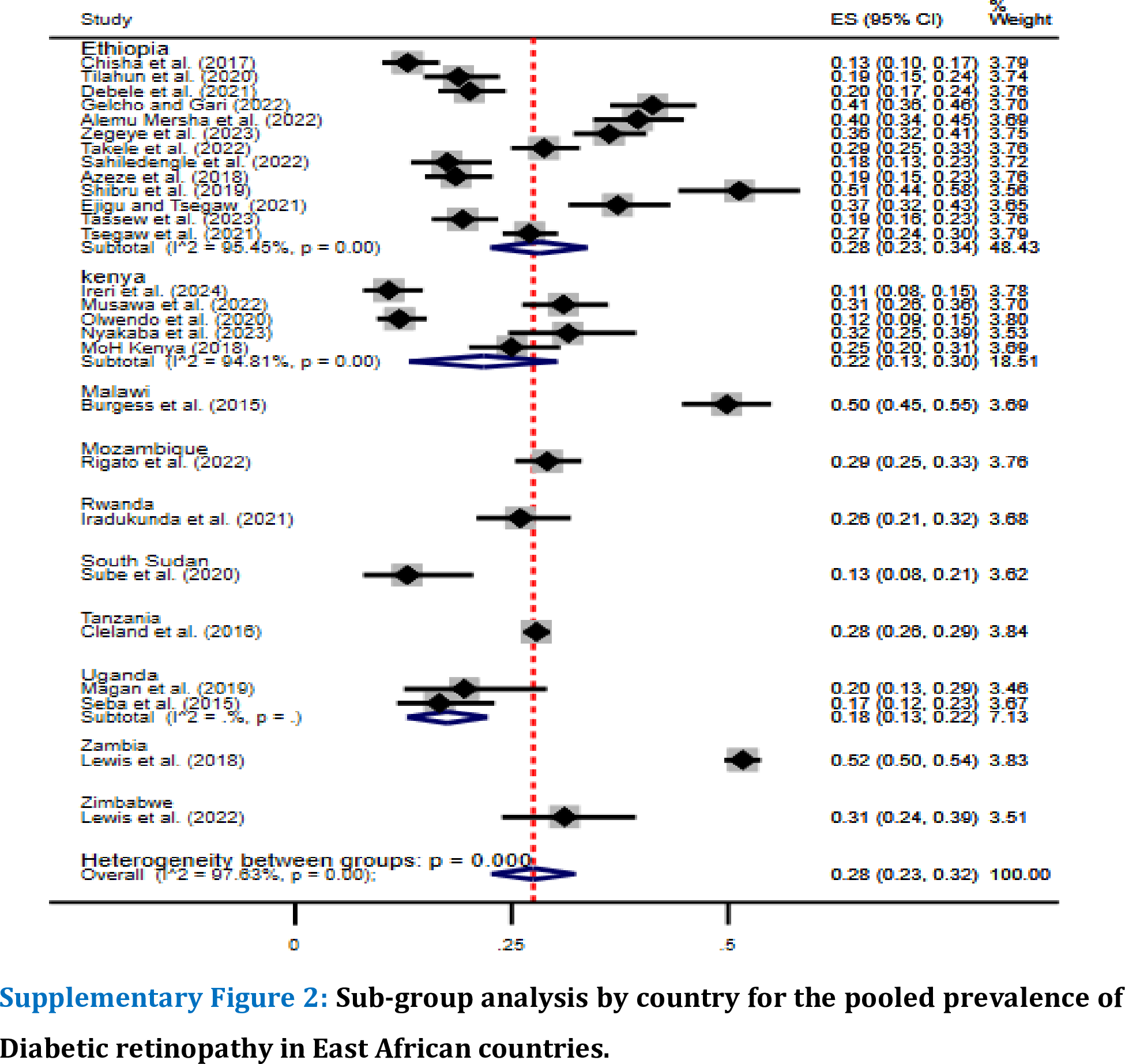

Supplement: S2 Fig — (TIF) [file pone.0316160.s005.tif]

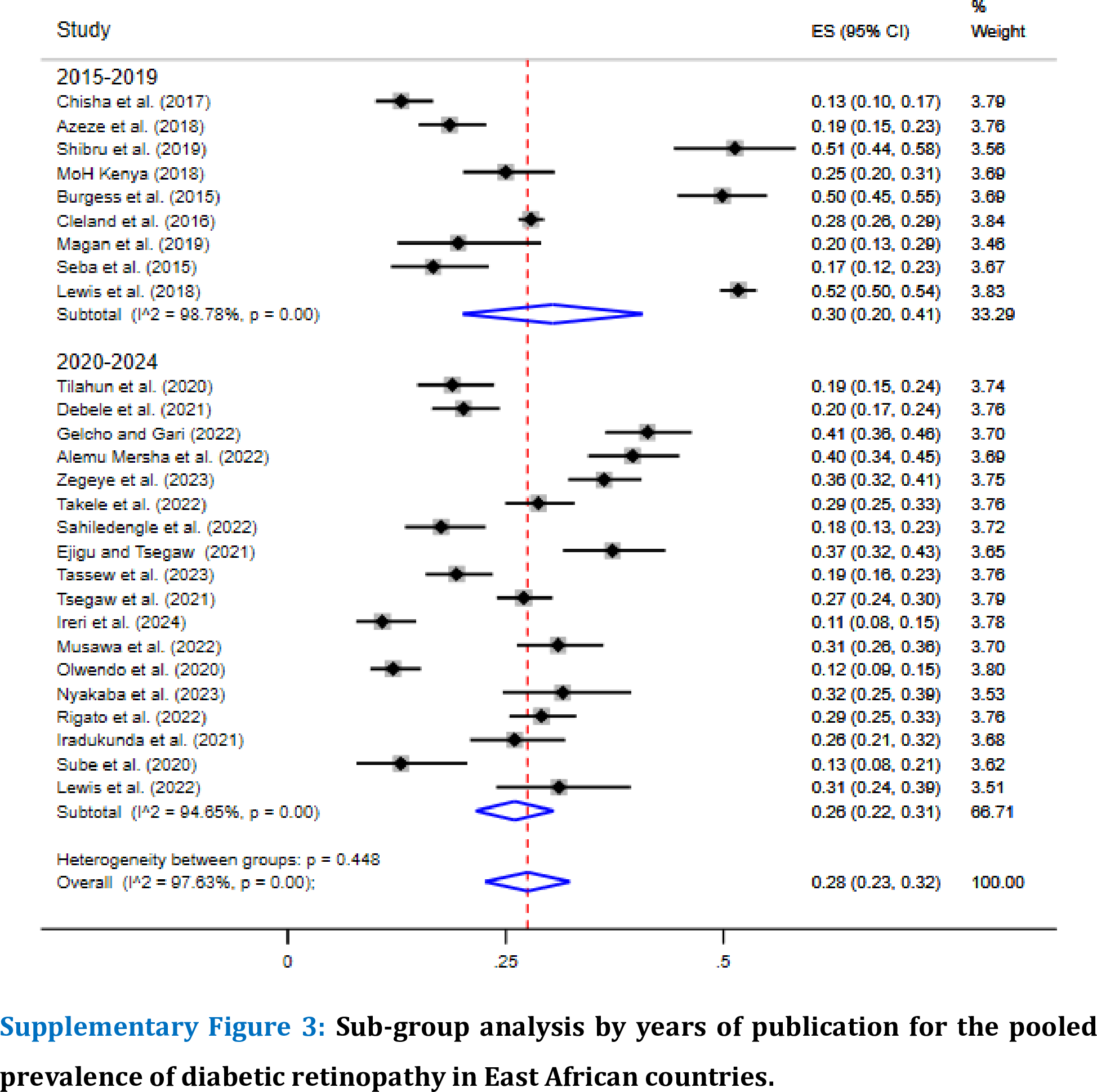

Supplement: S3 Fig — (TIF) [file pone.0316160.s006.tif]

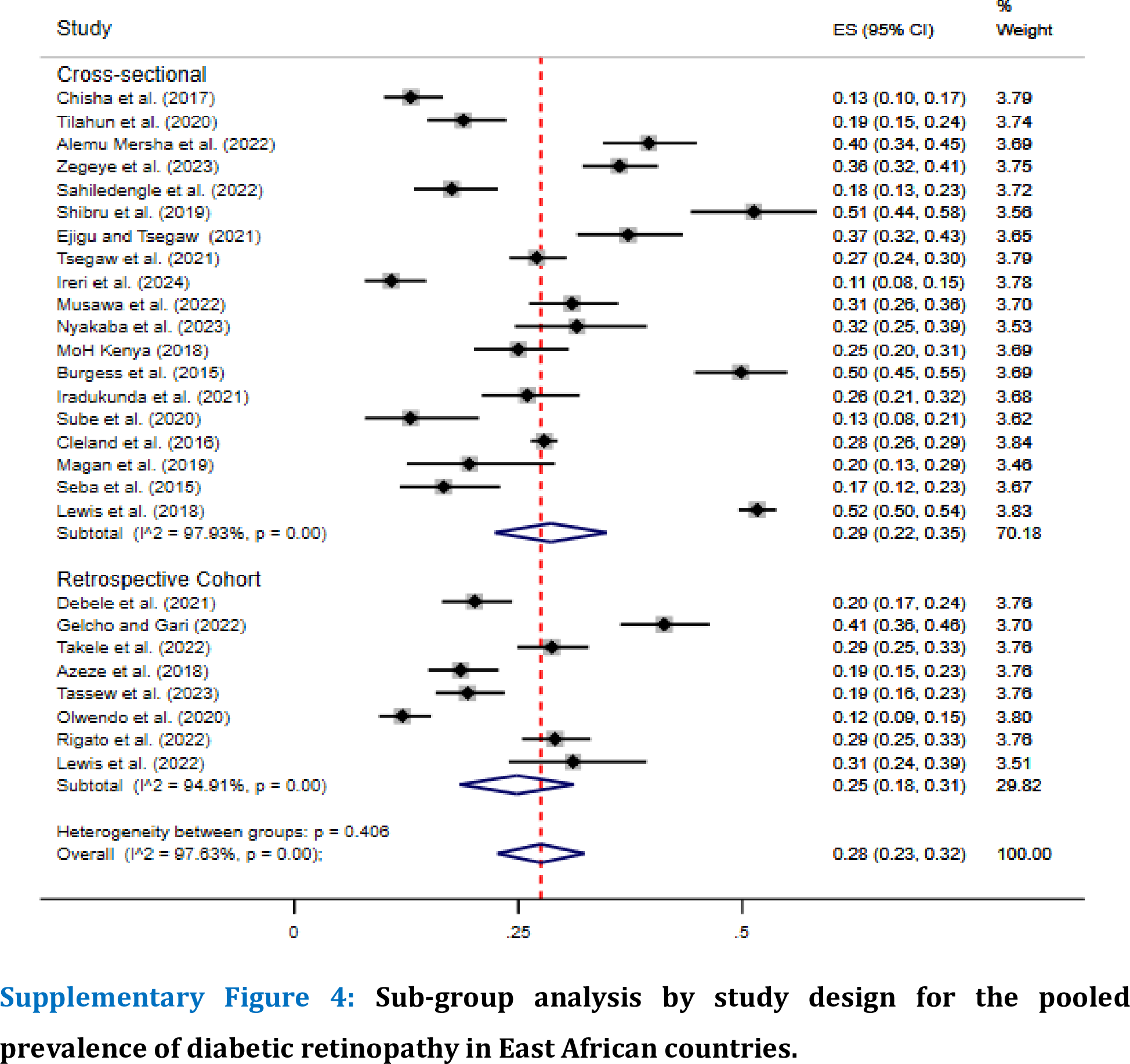

Supplement: S4 Fig — (TIF) [file pone.0316160.s007.tif]

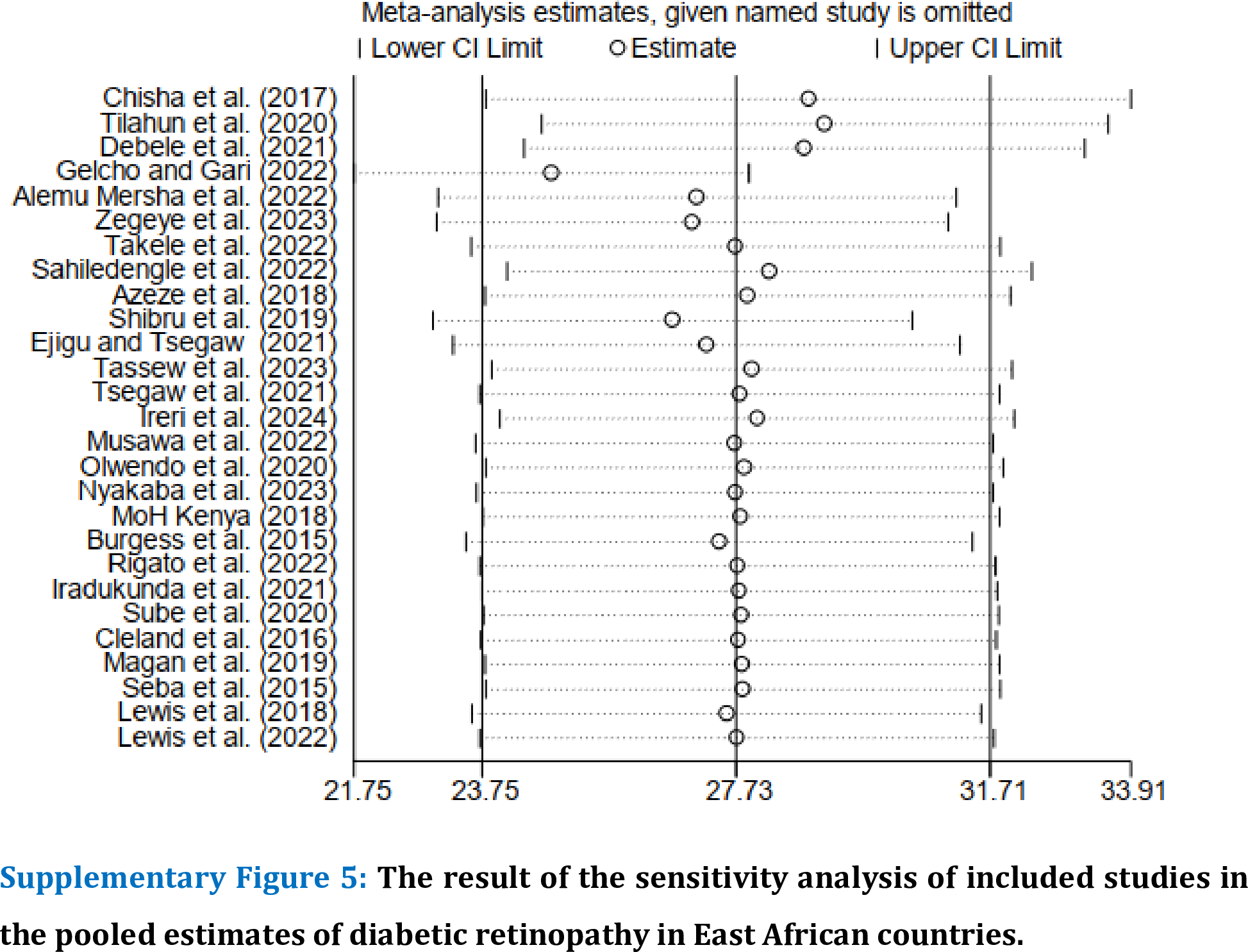

Supplement: S5 Fig — (TIF) [file pone.0316160.s008.tif]
